# Digital Technologies for Health Workforce Development in Low- and Middle-Income Countries: A Scoping Review

**DOI:** 10.9745/GHSP-D-18-00167

**Published:** 2018-10-10

**Authors:** Lesley-Anne Long, George Pariyo, Karin Kallander

**Affiliations:** a Independent Consultant, Washington, DC, USA.; b Department of International Health, Johns Hopkins Bloomberg School of Public Health, Baltimore, MD, USA.; c Division of Global Health, Department of Public Health Sciences, Karolinska Institutet, Stockholm, Sweden, and Malaria Consortium, London, UK.

## Abstract

The collection of journal articles, systematic reviews, and reports published over the last decade that attest to the potential of digital technologies to achieve health workforce improvements across all aspects of the health system is vast. As a capacity-building mechanism, digital technology has potential for low- and middle-income countries (LMICs) to support development of the health workforce, including those health workers based in remote or rural areas, to train, motivate, support, monitor, and pay them. The purpose of this scoping review to present, at a high level, the state of the evidence and best practices in digital strategies for human resources for health and to propose a roadmap for a research agenda to fill identified gaps in the evidence. A variety of peer-reviewed and gray literature sources were searched using selected key terms related to digital health and health workforce, limited to materials published from 2010 to 2018. More than 70 articles, reports, and blog posts were reviewed, with in-depth analysis of 29 articles. Findings show that a range of digital health solutions for health workforce development have been tested and used, such as for health worker training, provider-to-provider communication and professional networking, and supervision of and performance feedback to health workers. There is some evidence of improved efficiency and effectiveness, at least at the level of pilots or small-scale projects. There is, however, a growing urgency in global health to move beyond small-scale demonstration projects and to define the capital and recurring costs of implementation and scale up of digital health interventions, including the return on investment. The next frontier is to select, adapt, and implement at scale those digital health interventions for health worker development and management found to be most promising.

## Abstract (precis)

Digital health interventions have the potential to improve the health workforce by supporting training, supervision, and communication. More evidence is needed on the effectiveness of interventions implemented at scale, including the return on investment, the effect of government and donor policies on scale up, and the role of the private sector.

## INTRODUCTION

It is estimated that 2 billion more people will be born around the world over the next 35 years.^1^ The challenges facing fast-growing developing countries are serious and real, and the threat of global pandemics as well as increasing morbidity linked to non-communicable diseases (NCDs), such as cardiovascular conditions, cancers, and diabetes, will have dramatic and negative impacts on the health of populations globally.^2^^,^^3^

Evidence indicates that where frontline health workers are effectively trained and deployed, there is a reduction in maternal and child mortality, a reduction in the spread of HIV, TB, and malaria, and better management of chronic diseases.^4^ Coupled with effective support and supervision, relevant and regular training has the potential to build a health workforce that can extend the reach of quality health care, in particular to poor and marginalized populations.^5^ The Sustainable Development Goals (SDGs), especially SDG 3 (“ensure healthy lives and promote wellbeing for all at all ages”), recognize this and emphasize the need for a substantial increase in the recruitment, development, training, and retention of the health workforce, especially in least-developed countries.^6^^,^^7^ Yet gains in survival are being lost because of weak and fragmented health systems with overburdened and overstressed health workers—too few in number and without the necessary support, training, and supervision that they need.^8^

There is an emerging consensus among researchers, as well as among global leaders and donors, that digital health represents an opportunity for developing stronger health systems to ensure adequate service delivery by frontline health workers.^9^ Mobile connections globally now stand at 7.6 billion (with 4.7 billion unique users) and mobile broadband penetration has risen sharply in the last 10 years.^10^ Smartphone penetration is already 48%, and predictions are that there will be 5.6 billion smartphones by 2020 with 90% of users in low- and middle-income countries (LMICs).^10^

Digital health represents an opportunity for developing stronger health systems.

The collection of journal articles, systematic reviews, and reports published over the last decade that attest to the potential of digital technologies to achieve health workforce improvements across all aspects of the health system is impressive.^11^^,^^12^ As a capacity-building mechanism, digital technology—comprising the use of computers, tablets, and mobile phones—represents an opportunity for LMICs to train, motivate, support, monitor, and pay health workers. For example, digital health (a term used synonymously with e-health, mHealth, and mobile health in this article) offers a good opportunity to reach cadres such as community health workers (CHWs)^13^^,^^14^ or health workers in remote and hard-to-reach areas that are routinely underrepresented in human resources for health (HRH) information systems. A comprehensive World Health Organization (WHO) classification of digital health interventions^15^ highlights how technology potentially offers opportunities for intelligent devices to automate data collection, disease surveillance, and rapid testing as well as to improve accountability and strengthen the interconnections between frontline health workers, health facilities, and ministries of heath.^14^ However, despite this general understanding of its potential benefits, digital health at a national scale is still slow to becoming a reality in many LMICs and there remains limited use of technology for training, communication, and data sharing between health professionals.^10^

The purpose of this article is to provide an overview of the state of the evidence at a high level and best practices in digital strategies for HRH/health workforce development and to propose a research agenda where gaps in the evidence are identified. We used a scoping review approach, a form of knowledge synthesis, which aims to summarize and synthesize evidence to inform practice, programs, and policy and to provide direction to future research priorities.^16^^,^^17^

## METHODS

A scoping review was conducted using the framework recommended by Arksey and O'Malley^16^ and further developed by Colquhoun et al.^17^ The framework includes agreeing on clearly articulated questions, listing detailed inclusion criteria, designing a structured search process to locate and select relevant documentation, and agreeing on methods for critically appraising, extracting, and presenting the data.

This review was guided by the following 2 questions: “How are digital technologies potentially usable to improve health workforce capacity, confidence, and motivation?” “How can digital solutions improve monitoring of health staff performance across health system levels?” Documents included in the review were systematic reviews, thematic reviews, and other scoping reviews, as well as articles, reports, and blogs including published and unpublished (gray) literature. We excluded articles or reports that did not focus on LMICs, that considered digital health broadly (across several sectors), or that focused on digital financing or areas covered by the other articles included in this special supplement. We conducted searches on Google, Google Scholar, PubMed, the World Health Organization website, the Broadband Commission Working Group on Digital Health website, and the United States Agency for International Development website, limiting our search to materials published from 2010 to 2018. Search terms included “mobile health”, mHealth, “digital health”, eHealth, “ICT for health”, “frontline health worker”, “human resources for health”, “health workforce”, and “community health worker”.

Study selection involved applying the post-hoc inclusion and exclusion criteria based on the specifics of the research questions and on new familiarity with the subject matter after reading the studies. The most promising articles and reports were subjected to an in-depth analysis by the first author, who extracted relevant information using a descriptive analytical method for the contextual or process-oriented information.^17^ Examples of literature that were scrutinized included literature that featured successful implementation of digital health programs that provided effective health worker training using mobile devices, improved staff performance, and supported enhanced health service delivery. The descriptive data extracted were organized in conceptual categories to specifically shed light on the strength of current evidence for scaling programs using digital health technologies, as well as the documented weaknesses of that research.

## RESULTS

More than 70 reviews, articles, reports, and blog posts were analyzed. A more in-depth review of 29 articles (of which 8 were systematic, thematic, or scoping reviews) and reports informed the views and conclusions presented in this article.

### Why Digital Health?

The evidence suggests digital health has the potential to improve the efficiency of the health workforce, advance quality health services coverage, and enable better health outcomes.^18^ Digital health solutions that fit into existing health systems functions can support health systems strengthening objectives such as a well-performing health workforce; a functioning health information system; cost-effective use of medical products, vaccines, and technologies; and accountability and governance.^5^ Digital technologies can be used to train health workers, facilitate communication between them, provide job aids, and assist with supervision. In a review of the existing literature on use of digital health strategies by frontline health workers, DeRenzi et al.^19^ outlined 6 health systems functions that are typically addressed using digital technologies, including access to training and information for health workers, facilitation of communication between health workers, provision of job aids and decision support tools, and assistance with health worker supervision. The evidence to support these strategies is rapidly growing, though yet remains in early stages.^9^^,^^19^

Digital technologies can be used to train health workers, facilitate communication between them, provide job aids, and assist with supervision.

In relation to health workforce development specifically, there is a range of digital health interventions with some evidence of effectiveness, at least at the level of pilots or small-scale projects. Huang and colleagues state^20^:

There is a voluminous literature on a multiplicity of digital health innovations in middle and low income countries and in recent years a growing number of evaluation studies that have demonstrated beneficial impacts from these interventions.

Some of the benefits of introducing digital tools into existing systems for health workforce development include access to digital health training content, better support with professional networks, and an expanded role for health workers in active case detection using disease surveillance systems. For example, Cesario et al.^21^ find that:

Cellular phone use may contribute to the diagnosis and epidemiological surveillance of diseases in areas where physical access is difficult and/or which have limited human resources, medical, and technical equipment.

Furthermore, Nicole Ippoliti et al.^22^ indicate that:

Better data for decision making with virtually real-time reporting of services and commodities through a health management information system (HMIS) and logistics management information system (LMIS) improved provider capacity through continuous learning, digital provider tools, and mobile supervision [and] [i]ncreased transparency, efficiency, and accountability through digital financial services.

Digital tools can also promote continuing professional development to increase knowledge and skills as well as improve health workers' confidence in their day-to-day practice.^23^

Some of the recent research on digital health indicates promising impact and potential to scale, including:

- **Health worker training:** In a correspondence to the *Lancet Global Health*, O'Donovan and Bersin report on how mHealth strategies can be a low-cost, high-impact solution to mapping outbreaks and providing education for both health workers and the public.^24^ For example, mobile phones were used by the local community to send free text messages about Ebola to the Government in Sierra Leone, who then created heat maps that health workers could use to track the epidemic. In addition, digital strategies were successfully deployed for health care workers in Liberia where they downloaded onto their phones reference and training materials demonstrating the correct and proper use of personal protective equipment and safe injection and burial practices.
- **Provider-to-provider communication:** A number of digital health applications have been designed as tools to increase communication and professional networking to support health workers, with many concluding that basic mobile phone use along with SMS capabilities can improve CHW efficiency while potentially reducing overall program costs.^25^ In Malawi, CHWs were provided with SMS capabilities to report medical supply shortages and communicate both general information and specific information about patients with emergencies.^26^ The average cost per communication was about 5 times less expensive using SMS than in areas without SMS service, and reporting was significantly faster. Similarly, in another study using SMS for CHWs providing tuberculosis (TB) and HIV care in Malawi,^27^ communication between CHWs and the hospital staff freed up over 2,000 hours of hospital staff time, which resulted in expanded health care delivery capacity and a greater number of TB patients starting treatment.
- **Human resource management:** Supervision of and performance feedback to health workers is critical to maintaining their motivation, improving their performance, and retaining them in their posts.^28^ Digital tools can provide a mechanism for delivering supervision to remote health workers who often work in isolation with little and infrequent support.^29^ In sub-Saharan Africa and in rural areas more generally, where health worker coverage is the lowest, digital health offers the promise of maximizing health care worker impact and efficiency.^25^^,^^30^ In India, for example, CHWs working to reduce malnutrition in their communities who received performance feedback and supportive supervision via mobile phone calls were shown to have improved motivation and performance compared with their motivation and performance before the program.^31^ The study suggests that regardless of the performance information disclosed, calls can improve performance due to elements of supportive supervision encouraging CHW motivation. There is also evidence that establishing a high-quality human resources information system can support health workforce management and efficient targeting of limited domestic resources.^12^

Other literature pointed to gaps where additional research is needed, including:

- **Total cost of ownership and implementation**: Although digital health interventions offer the prospect of long-term cost savings, they usually require significant investments upfront as well as regular expenditure on training and maintenance. There has been some research on total cost of ownership^32^^,^^33^ but mostly with the purpose to develop an advocacy tool to highlight how a certain device or particular software provides the most cost-effective way to deliver digital health programs. There is less evidence on the capital and recurring costs needed to implement and maintain, let alone scale up, a digital health program.
- Linked to this is the need to demonstrate the **return on investment** (ROI) in digital health. Government (largely via donor funding) is likely to be the largest funder of digital health initiatives in developing countries. Digital health stakeholders need to stimulate government investment by demonstrating how digital health solutions help address national health care issues. This is a critical knowledge gap; without longitudinal evidence and in the face of limited examples of digital enterprise architectures delivering better health outcomes, governments and global health donors remain unlikely to prioritize investments into large-scale HRH digital health programs.

### Strength of the Evidence

As of 2015, there were more than 6,000 peer-reviewed or gray literature articles or documents on digital health.^10^ Evidence on the actual impact of digital health on population health outcomes is still largely lacking in the peer-reviewed literature.^34^^,^^35^ Bergmo explains^35^:

Despite promising results, more evidence is needed on the cost implications of digital health and the degree to which it can improve health outcomes over the short and long term. While there is anecdotal evidence that digital health can bring health benefits, the lack of sufficient rigorous clinical evidence and large-scale studies to confirm this claim is a barrier to investment.

It is this lack of evidence that presents one of the key challenges to moving digital health approaches from pilot projects to national programs while properly engaging health workers and communities in the process. A common feature emerging from literature published in the last 5 years (i.e., once the initial excitement of digital health innovation began to settle into a valley of ‘reality-on-the-ground’) is that a major weakness of digital health interventions is that the claimed benefits are unclear and long-term results remain uncertain.^36^ David Novillo-Ortiz, coordinator of the Regional eHealth Program of the Pan American Health Organization/WHO, is quoted in the Broadband Commission for Sustainable Development^10^ as saying:

The big challenge is to ensure the sustainability and continuity of digital health initiatives, whose benefits can sometimes only become apparent after ten to fifteen years. And to accomplish this, it is fundamental to promote the production of scientific evidence to raise awareness among decision-makers about the importance of investing in eHealth.

While studies may be successful at a small or moderate scale, large-scale or nationwide coverage of digital health interventions to support health workforce development is still rarely reported in the literature. Though rife with a descriptive analysis of the potential of such interventions on health care outcomes, few have empirically assessed the incremental effectiveness of such interventions on health care coverage, utilization, efficiency, quality, or outcomes.^37^ The speed of innovation, including the constant evolution of platforms, the broad range of initiatives and tools, and the heterogeneity of reporting have also made it difficult to uncover and synthesize how digital health tools might be effective.^38^ Until the WHO published the classification of digital health interventions in 2018, an absence of shared language and approach to describe digital health interventions made it challenging to compare results across programs.^5^ Furthermore, economic evidence for investing in digital health is limited. While often reputed to be cost-effective or cost-saving, the strength of the evidence supporting this assertion is typically considered insufficient to attract significant investment. In a systematic review of the economic evaluations of digital health, unknown cost-effectiveness was listed as one of the top 6 barriers to digital health implementation and a key factor in limited digital health policy investment.^39^

## DISCUSSION

Digital technologies, when focused on delivery of care, training, staff performance, and monitoring, have the potential to improve health workforce capacity, confidence, and motivation. With partnerships forming between governments, technologists, NGOs, academia, and industry, the literature suggests there is increasing potential to improve health services delivery and support health workforce development by using digital health in LMICs. By harnessing the increasing presence of mobile phones among health workers, digital health can be used to deliver increased and enhanced health care services to individuals and communities while helping to strengthen health systems.^14^

Digital health use at national scale is slow to becoming a reality in many LMICs despite the general understanding of its potential benefits,^6^ and there remains limited use of technology for communication and data sharing between health professionals.^40^ Although digital technology can be a highly effective delivery system for national health systems, it, so far, has not been deployed as widely as technologists have anticipated. The research community has struggled to create an evidence base for decision makers to confidently expand digital health pilots to a provincial or national level.^34^^,^^35^

Digital health use at national scale is slow to becoming a reality in many LMICs.

There are several well-catalogued reasons for this general failure of pilots being taken to scale. The research points to the lack of coordinated approaches to digital health investments, leading to a fragmented digital health landscape, with multiple pilot interventions or short-term projects that do not include planning for scale or sustainability. Furthermore, traditional development funding flows are often disease- or country-specific, limiting adaptation, inhibiting data sharing, and resulting in inefficiencies.

In addition, until recently, there have been few agreed digital health commons, hubs of best practice, or standardized guidelines to guide implementers and governments in how to develop scalable, interoperable, and sustainable digital health systems for health workforce development. There is some progress in this respect, however: broad consensus around the shared values of digital development are enshrined in the Principles for Digital Development,^41^ and guidance has been developed by WHO, the International Telecommunication Union, and others on how to develop and maintain digital health systems at scale.^42^^–^^44^ Nonetheless, it remains challenging for donors to align funding and activities to support governments to create effective digital systems that are sustainable beyond the term of any one project or program.^45^ This despite recommendations urging greater collaboration around investment in digital health systems at national level^46^ and commitment from over 20 donor organizations to align around investments in digital health.^47^ In fact, it is still not unusual for digital health interventions to be developed and operated without any involvement of national governments. In addition, digital health solutions may be introduced by organizations without clear knowledge of the state of affected countries' information technology infrastructure at national and decentralized levels, making implementation of solutions inappropriate and, in the worst cases, resource-draining.^45^

### A Roadmap for Research

Key areas of digital health research needed, as highlighted by the literature, include the following:

**Value of scaling up digital health approaches to HRH management and support**. Given the clear message from the literature that the case for investing in, and scaling up, digital health remains to be made, areas for additional inquiry include how digital technologies could be leveraged to expand the reach of performance management and timeliness of feedback for CHWs and other frontline health workers not currently being reached, timely updates of data for workforce planning and projections, cost-effectiveness of continuing education using digital health versus traditional approaches, and updating training needs assessments by regularly reaching out to health workers to ensure training content is well tuned to actual needs on the ground, among others.

**Better evidence on the ROI of digital health**. This could be addressed in the short term by economic modeling and research into the ROI of digital approaches in other sectors (e.g., the financial and agriculture sectors). In the longer term, the need for better ROI evidence could be addressed by working with governments implementing regional or national digital health strategies, such as the East African Community Digital Health Roadmap (Digital REACH) and the Tanzania Roadmap for Digital Health.

**Effect of current donor and government procurement policies on scale up of digital health technologies for health workforce development**. Although technology used in health worker training, supervision, and data collection programs is often designed to handle a large volume of information, it is often procured on the basis of single health issues and/or limited to specific geographic regions within countries. Procurement on this basis does not take advantage of the inherent scalability of the technology.

**Role of the private sector and philanthropists in digital health.** Despite calls for greater engagement of and investment by the private sector and philanthropic investors in taking digital health technologies to scale in a sustainable way, there are few examples of this kind of partnership happening at scale (or at all). This links back to ROI—without a strong business case for investment in national-level digital health infrastructure, the private sector will not move beyond the transactional corporate social responsibility approach to one based on long-term relationship building and investment. More research is needed into the role of the private sector, taking into account the diverse nature of that sector, and philanthropists in developing and implementing digital health strategies and how to engage them more effectively in this space.

The authors propose that an implementation research perspective, involving an iteration of learning by doing, analysis, and selection of the most promising approaches, will help document additional lessons learned and answer some of the remaining critical questions.^48^ An approach of ‘more of the same’ will result in the continued lack of evidence and represent a lost opportunity to leverage the potential capabilities that improved digital technologies can contribute to the health sector.

### Strengths and Limitations

The scoping review approach we used has some strengths and limitations. On the one hand, it allows for presentation, in one publication, of the state of evidence on a particular issue that is useful for policy makers wishing to obtain a high-level view of the topic. But in doing so, it misses out on the type of details that some may want to see. As stated earlier, our main purpose was to present a high-level view of the state of evidence for using digital health technologies for health workforce development and to propose areas for further inquiry in the form of a research agenda that donors and researchers may wish to consider.

## CONCLUSION

There is an urgent need to provide the evidence that will support a move beyond small-scale, demonstration projects to selecting, adapting, and implementing at scale those digital health interventions found to be most promising. More research is needed to respond to questions about how governments can develop their digital health leadership and harness the dramatic penetration of mobile and web technology to help improve the lives of their citizens. There needs to be a better understanding of how governments can motivate private- and public-sector staff to deliver tangible growth and security through digital health solutions that harness the advances in technology domains such as network speed and efficiency, cloud computing, data connectivity, and data analytics to improve health workforce capacity and improve access to and quality of health care services. An implementation research perspective will help uncover answers to these questions and document additional lessons learned. An ongoing reluctance to examine these bigger issues will lead to lost opportunities to leverage the remarkable capabilities that improved digital technologies have brought to the health sector.

## Notes

**Peer Reviewed**

**Funding**: This work was made possible by a grant from the Aetna Foundation, with support from the World Health Organization and The Johns Hopkins University Global mHealth Initiative.
